# Expectancy of Stress-Reducing Aromatherapy Effect and Performance on a Stress-Sensitive Cognitive Task

**DOI:** 10.1155/2015/419812

**Published:** 2015-01-31

**Authors:** Irina Chamine, Barry S. Oken

**Affiliations:** ^1^Department of Behavioral Neuroscience, Oregon Health & Science University, Mail Code CR120, 3181 SW Sam Jackson Park Road, Portland, OR 97239, USA; ^2^Departments of Neurology, Behavioral Neuroscience and Biomedical Engineering, Oregon Health & Science University, Mail Code CR120, 3181 SW Sam Jackson Park Road, Portland, OR 97239, USA

## Abstract

*Objective*. Stress-reducing therapies help maintain cognitive performance during stress. Aromatherapy is popular for stress reduction, but its effectiveness and mechanism are unclear. This study examined stress-reducing effects of aromatherapy on cognitive function using the go/no-go (GNG) task performance and event related potentials (ERP) components sensitive to stress. The study also assessed the importance of expectancy in aromatherapy actions. *Methods*. 81 adults were randomized to 3 aroma groups (active experimental, detectable, and undetectable placebo) and 2 prime subgroups (prime suggesting stress-reducing aroma effects or no-prime). GNG performance, ERPs, subjective expected aroma effects, and stress ratings were assessed at baseline and poststress. *Results*. No specific aroma effects on stress or cognition were observed. However, regardless of experienced aroma, people receiving a prime displayed faster poststress median reaction times than those receiving no prime. A significant interaction for N200 amplitude indicated divergent ERP patterns between baseline and poststress for go and no-go stimuli depending on the prime subgroup. Furthermore, trends for beneficial prime effects were shown on poststress no-go N200/P300 latencies and N200 amplitude. *Conclusion*. While there were no aroma-specific effects on stress or cognition, these results highlight the role of expectancy for poststress response inhibition and attention.

## 1. Introduction

Stress-management approaches can ameliorate negative consequences of stress on health and cognition [1]. Aromatherapy, therapeutic use of inhaled essential oils, is a popular stress-reducing approach due to low side effects [2, 3], but its effectiveness is questioned. Despite research supporting aromatherapy effectiveness, the existing evidence is not convincing, according to a comprehensive review, due to the poor quality of previous studies [4]. Small samples, mostly subjective outcomes, and inadequate controls and blinding are the major criticisms about previous research [4, 5]. Lack of convincing evidence for aromatherapy actions is also linked to insufficient understanding of aromatherapy mechanism.

The criticisms raised by the previous reviews were addressed by evaluating stress-reducing properties of lavender (*Lavandula angustifolia*) essential oil in a more rigorous study. Lavender, popular for relaxation and stress reduction [6], has been previously shown to improve cognition [7] and decrease agitation [8, 9], stress [10–13], and anxiety [14–17]. However, some studies demonstrated no specific lavender effects concluding that the changes after aromatherapy exposure occur solely due to expectancy of improvement [18, 19] and considered aromatherapy an “ineffective treatment but an effective placebo” [20]. To evaluate this idea, the role of expectancy in aromatherapy actions was the focus of our investigation.

As a part of a larger trial assessing aromatherapy effects on physiological and cognitive functions [21], this study was rigorously designed and used objective outcomes, two different controls, and assessors blinded to the groups assignment. Stress-reducing lavender aromatherapy properties were evaluated by testing lavender effects on performance on a cognitive task subserved by executive function especially vulnerable to stress [22]. We used a visual go/no-go (GNG) task, requiring responses to some target stimuli (go conditions) and withholding responses to other target stimuli (no-go conditions). Inhibitory control assessed by the GNG task is a central component of executive function [23] and can be assessed through event related potentials (ERPs) by evaluating no-go N200 and P300 [23]. Typically used in relation to processing salient stimuli mediated by attention, N200/P300 components during the GNG represent response inhibition subprocesses vulnerable to acute stress [24]. N200 occurring approximately 200 ms after stimulus onset [25] has enhanced amplitude after stress [24]. Also, N200 of larger amplitude and shorter latency distinguishes good inhibitors from the bad ones [26]. P300 occurring between 250 and 650 ms after stimulus onset relates to stimulus classification speed and attention resource allocation [27].

In this study, we assessed how aromatherapy exposure during stress affects poststress GNG performance and ERPs. We also evaluated how the poststress GNG and ERPs are influenced by expectancy enhanced by a suggestion of powerful stress-reducing properties of the assigned aroma.

## 2. Methods

### 2.1. Participants

Eighty-one adults (*M*_age_ = 58.2, 79% females), a subset from a larger trial evaluating stress-reducing aromatherapy effects (clinical trial ID number NCT01307748), were recruited from the community through advertisements. Eligibility criteria were assessed by a phone interview and included (1) age between 50 and 85 years; (2) good physical and cognitive health (score ≥31 on the Modified Telephone Interview for Cognitive Status [28]); (3) score ≥9 on the Perceived Stress Scale [29] indicating presence of stress; (4) no medications affecting CNS or physiologic measures; (5) healthy olfactory function; and (6) nonsmoking. The study was approved by the Oregon Health & Science University Institutional Review Board.

### 2.2. Procedure

After screening, each participant was randomized to an aroma group (lavender, coconut, or water) and a prime or no-prime subgroup as described below.

#### 2.2.1. Aroma-Based Groups

Each participant experienced one aroma during the study. To enhance expectancy and blinding, participants were told that the purpose of the study was to understand how different aromas affect the body and to test effects of several different aromas on stress responses. The participants were also told that the aromas were diluted and might not be perceptible but remain effective even without the obvious smell. The aromas tested in the study included detectable putative stress-reducing, detectable placebo, and undetectable placebo ones.

Detectable placebo aroma served as a control for the presence of a pleasant smell without stress-relieving pharmacologic properties to distinguish the effects arising from a pleasant smell from those arising from specific pharmacologic properties of the essential oil tested. Undetectable placebo aroma was used as a nonactive control without smell.

For the experimental stress-reducing aroma, a drop of lavender (*Lavandula angustifolia*) essential oil (Mountain Rose Herbs, Eugene, OR) was diluted in 15 mL of grapeseed carrier oil (Now Foods, Bloomingdale, IL).

For the detectable placebo aroma, a teaspoon of virgin coconut (*Cocos nucifera*) base oil (The Ananda Apothecary, Boulder, CO) was diluted in 15 mL of grapeseed carrier oil (Now Foods, Bloomingdale, IL) and mixed until solution appeared clear.

For the undetectable placebo aroma, distilled water was used giving the appearance of essential oil without producing odor or stress-reducing effects.

#### 2.2.2. Aroma Administration

During the visit, three drops of the aroma solution were placed on a 5 × 5 mm cotton pad. The pad attached by transparent odor-free tape came to the midpoint between the participant's nose and upper lip and remained attached until the study completion.

#### 2.2.3. Prime-Based Subgroups

In addition to aroma group, each participant was randomized into a prime subgroup to assess expectancy effects, with half of the participants in each aroma group receiving a prime and the rest receiving no prime.

Participants in prime subgroup, just prior to aroma exposure, received a card reading: “You are about to experience a powerful relaxing and stress-reducing aroma. To experience it best, please close your eyes and inhale the aroma deeply. You may or may not perceive this aroma. This aroma is known to provide a profound relief from stress whether or not people are able to perceive it.”

Participants in no-prime subgroup received a card reading: “You are about to experience an aroma that may or may not help reduce your stress level. Please close your eyes and inhale the aroma deeply. You may or may not perceive this aroma. Aromatherapy can be effective whether or not people are able to perceive aromas.”

All randomizations were completed by nonblinded researchers using a computerized adaptive randomization procedure [30] aimed at balancing aroma groups on distributions of age, gender, and stress score obtained at screening. Nonblinded researchers also performed debriefings.

#### 2.2.4. Blinding

All study visits were conducted by an assessor blinded to the participants' aroma and prime assignment. To avoid perceiving odors, disposable active carbon nose filters (Breathe-Ezy Nasal Filters, Henderson, NV) were used.

### 2.3. Laboratory Visit

All visits started between 12 pm and 12:30 pm to minimize circadian effects.

During the visit, participants signed the consent form and confirmed their eligibility by screening for general anosmia with a Quick Smell Identification Test (Sensonics Inc., Haddon Heights, NJ) [31]. Next, participants were fitted for ERP recording as described below. After a 30–40 minute adaptation period that allowed participants to get used to the laboratory setting, the participants started the study procedures involving a baseline assessment, stress battery, and poststress assessment (Table 1). Participants completed self-report and cognitive measures at baseline. Then each participant received a card with or without the prime and began inhaling the assigned aroma. Five minutes afterwards, participants underwent a stress battery followed by the poststress assessment similar to the baseline assessment. The visit took 4 hours. After completing procedures, the participants were debriefed and reimbursed $10.00 per hour.

### 2.4. Stress Battery

The stress battery consisted of emotional, physical, and mental stressor. Several stressors of different types were used to ensure that participants are stressed by some or all of the presented stressors. The stressors used in the study were chosen because they are tolerated well by human subjects and have been previously demonstrated to be effective in eliciting a stress response and influencing physiologic, endocrine, and cognitive functions that were assessed in the larger trial.

*Physical stressor* involved the cold pressor task [32] that required keeping a hand in ice-cold water as long as it is tolerable or until 3 minutes elapsed.

*Emotional stressor* was a 5-minute slideshow of unpleasant images from the International Affective Picture System [33]. Images with valence ≤5 and arousal level ≥5 were presented. To ensure subjects' attention, participants were instructed to push a button when snake images appeared.

*Mental stressor* involved the Montreal Imaging Stress Task (MIST) composed of computerized mental arithmetic with a built-in failure algorithm and social evaluative threat component [34]. The MIST software was generously provided by Dr. Jens Pruessner of McGill University.

### 2.5. Self-Reports

Participants answered questions about demographics and aromatherapy use.

*Perceived stress scale (PSS)* [29] was used to assess perceived stress level in the previous week. During the screening process, we used the cut-off scores ≥9 because this score represented the mean stress level observed in healthy adults of similar age estimated from previous studies conducted in our laboratory. Recruiting subjects with perceived stress level that is at or above estimated average stress level for the targeted population was done to ensure enrollment of subjects most sensitive to laboratory stressors and thus most likely to benefit from any potential stress-reducing aromatherapy effects.

*Expectancy of aromatherapy effect* was assessed with visual analog scales (VAS) ranging from 0 to 100 mm. Participants rated expected aromatherapy effect on stress (from decreased to increased stress) and general aromatherapy effect (from overall negative to overall positive effect).

### 2.6. Stress-Related Measures

#### 2.6.1. Salivary Cortisol

Cortisol samples were collected using Sarstedt Salivettes (Sarstedt AG & Co, Nümbrecht, Germany). Samples were stored in a refrigerator shortly after collection and were centrifuged following the visit completion and stored at −80 F prior to processing by the Oregon Clinical Translational Research Institute Core Laboratory. Saliva samples from each subject at baseline, after stress battery, and during the post stress assessments were run in the same assay batch.

Values for cortisol levels were obtained using a commercially available ELISA kit (Salimetrics, State College, PA 16803).

*Subjective stress* was rated with a VAS ranging from 0 (no stress) to 100 (extremely stressed).

*The positive and negative affect schedule (PANAS)* [35] was used to assess positive and negative affect at the time of the assessment. Item for alertness was used as a proxy measure of alertness.

### 2.7. Cognitive Measures

*Simple reaction time (SRT)* test was used to evaluate processing speed [36]. Participants pushed a button as quickly as possible when each of twenty-four stimuli (letter “O”) appeared on the computer screen.

*Go/no-go (GNG) task* included 200 uncued stimuli (80% letter “O” go stimuli, and 20% letter “Q” no-go stimuli) presented for 100 ms on the screen at pseudo-random order with an intertrial interval varying randomly between 1000 and 1300 ms.

Computer tasks were presented using EPrime software version 2 (Psychology Software Tools Inc., Sharpsburg, PA).

### 2.8. ERPs

EEG was recorded from 32-channel array (10/20 system) using BioSemi Active Two equipment (BioSemi B.V., Amsterdam, Netherlands). The electrooculogram (EOG) was recorded from the electrodes placed above the left external canthus and below the right external canthus. The single-ended signals were converted to differential signals offline with electrodes from right and left hemisphere referenced to the average of both mastoid electrodes. The EEG recordings were monitored to adhere to the offset recording standards of the BioSemi Active Two system. The sampling rate was 1024 Hz.

#### 2.8.1. ERP Processing

All processing was completed offline using BrainVision Analyzer 2.0 (Brain Products GmbH, Gilching, Germany). Average mastoid reference EEG was filtered offline from 0.1 to 70 Hz with a 60 Hz notch filter. Ocular artifacts were removed using independent component analysis [37], and epochs containing other artifacts were removed manually. Segments included 100 ms of activity prior and 1000 ms of activity following the stimulus onset. Each stimulus type data from correct trials was averaged. N200 was measured relative to baseline as the most negative peak occurring between 170 and 350 ms after stimulus onset. P300 was measured relative to baseline as the most positive peak occurring between 250 and 600 ms after stimulus onset. N200/P300 peak latencies and amplitudes were assessed with BrainVision Analyzer semiautomatic detection function and checked manually.

### 2.9. Statistics

To assess normality we examined histograms and boxplots and, if needed, used the log, square root, or Box-Cox transforms.

One subject lost GNG data due to computer problems. Some ERP data were lost due to severe artifact contamination (*n* = 7). The data loss was approximately equal in different groups and subgroups. Differences in sample sizes for different measures reflect these data loss issues.

### 2.10. Primary Analyses

#### 2.10.1. Behavioral Data

Repeated measures analyses of variance (rmANOVA) with time (baseline versus poststress), aroma (lavender, coconut, and water), and prime subgroup (prime, no-prime) were used to assess effects and interactions in behavioral performance. If groups or subgroups differed on any baseline variable affecting cognitive performance, that variable was used as a covariate in the subsequent analyses.

#### 2.10.2. ERPs

N200/P300 components are typically frontocentrally maximal for no-go stimuli and centroparietally maximal for go stimuli [38]. Visual inspection of ERP waveforms suggested the most robust waveforms at Cz; therefore, the analyses were conducted with the Cz data. rmANOVA with time (baseline versus poststress), stimulus type (go versus no-go), aroma (lavender, coconut, and water), and prime subgroup (prime, no-prime) were performed to assess interactions in N200 and P300 amplitude and latency. Additionally, no-go trials were the main focus of the follow-up analyses when an interaction was suggested. To avoid overinflating Type I error due to multiple comparisons, a Bonferroni correction was used to determine significance of each statistical family (aroma effects, prime effects). *P* values below .0125 were considered significant and those below .05 were considered trends.

### 2.11. Secondary Analyses

Bivariate Pearson correlations were performed to evaluate relationships among poststress cognitive performance and ERP variables as well as variables associated with expectancy and stress. Due to exploratory nature of the analyses, no multiple comparisons adjustments were used and significance level was at .05.

## 3. Results

### 3.1. Baseline Characteristics

The aroma groups and prime subgroups were well matched on most variables (all *P*'s > .05) as presented in Table 2. However, the participants in no-prime group had more education compared to prime group (*P* = .04). Therefore, education was a covariate to the behavioral data analyses.

### 3.2. Stressors Effectiveness

As planned, following the stressor there was a significant increase in subjective stress and decrease in positive affect [39]. Furthermore, changes in stress-related biomarkers (e.g., cortisol) and SRT reaction time indicative of an increased stress were observed [21]. These findings confirm that the stress battery affected participants' physiological and cognitive functioning.

### 3.3. Primary Analyses

#### 3.3.1. Behavioral Results

Groups or subgroups did not differ on baseline GNG performance (Tables 3(a) and 3(b)) or ERP measures, all *P*'s > .05.

Error rates remained stable for all participants regardless of the time point and group/subgroup, all *P*'s > 0.10. The GNG paradigm often fails to differentiate performances based on error rates when healthy adults are tested [23]. Therefore, the variable of interest was the median reaction time (RT) during the correct go trials.

#### 3.3.2. GNG Median RT for Go Trials

rmANCOVA (covariate: education) indicated shorter median RT to poststress go stimuli, *F*(1,75) = 4.82, *P* = .03, a common occurrence due to learning. Further, a time by prime interaction was shown, *F*(1,75) = 4.52, *P* = .037. The follow-up analyses showed that, regardless of the aroma present, participants in the prime subgroup had a significant poststress decrease in their median RT beyond general learning effects, *P* < .001, unlike the participants in the no-prime subgroup, *P* > .05. No significant effect of aroma was observed, *P* > .05.

### 3.4. ERP Results

**Figure 1:**
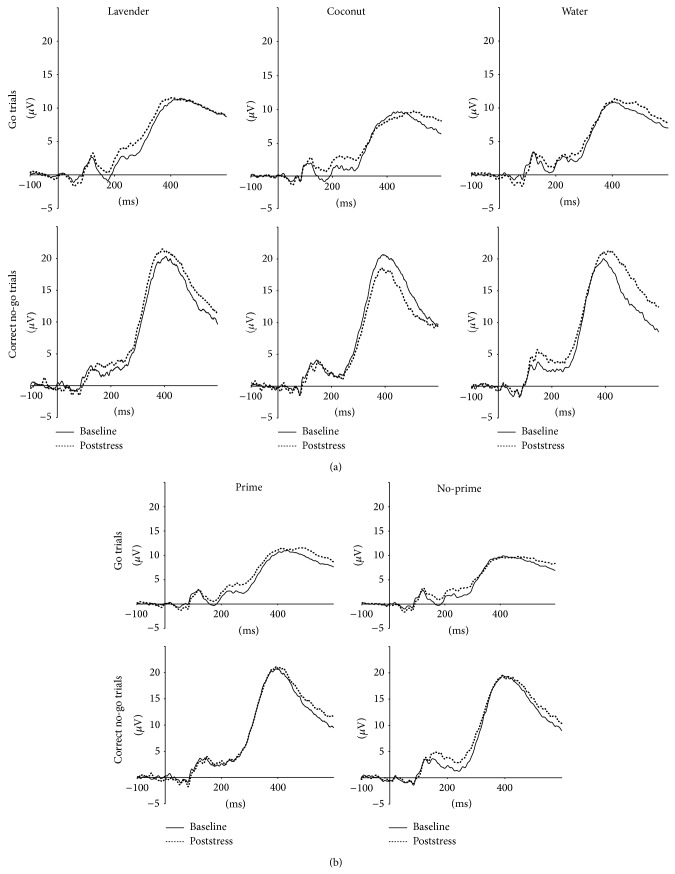
(a) ERP waveforms for aroma groups, (b) ERP waveforms for prime subgroups. Note: all data presented are from the Cz channel. Positive ERP values are plotted upwards.

Figures 1(a) and 1(b) display ERP waveforms for aroma groups and prime subgroups. As expected, distinct GNG effect was observed in P300 amplitude with no-go trials eliciting larger amplitude than go trials, *P* < .001. Similarly, main effects of a stimulus type were shown for N200 and P300 latencies, with no-go trials associated with shorter latencies for both components, *P*'s ≤ .01. Time effects were noted for N200 and P300 amplitudes. Specifically, there were lower N200 amplitudes for poststress go trials compared to baseline, *P* = .026, and reduced difference in P300 amplitude between poststress go and no-go trials compared to baseline. These time effects are likely due to stress and fatigue. These results are consistent with typically observed patterns for these ERP components [26, 40].

### 3.5. Group/Subgroup Differences

The following analyses assessed ERP differences between aroma groups and prime subgroups with the main focus on no-go stimuli. Generally, decreased latencies and increased amplitudes are indicative of more preserved cognitive function.

### 3.6. Aroma Effects

#### 3.6.1. N200 Amplitude

A trend for the time × stimulus type × aroma interaction, *F*(2,66) = 3.49, *P* = .036, suggested divergent ERP patterns between baseline and poststress for go and no-go stimuli depending on the aroma group. No additional significant interactions or aroma-specific effects were observed in follow-up analyses.

No effects were found for other ERP components.

### 3.7. Prime Effects

#### 3.7.1. N200 Amplitude

A significant time × stimulus type × prime interaction, *F*(1,66) = 7.59, *P* = .008, indicated divergent ERP patterns between baseline and poststress for go and no-go stimuli depending on the presence or absence of the prime. Follow-up analyses revealed a trend for a time × prime interaction, *F*(1,68) = 5.11, *P* = .027, for no-go trials. Specifically, participants receiving no prime displayed a lower poststress no-go N200 amplitude compared to baseline, *F*(1,33) = 7.68, *P* = .009, while participants receiving a prime had no change in poststress no-go N200 amplitude.

#### 3.7.2. N200 Latency

A trend for a three-way interaction for time × stimulus type × prime, *F*(2,66) = 4.77, *P* = .033, was observed. Specifically, participants receiving a prime had a trend for a poststress decrease in no-go N200 latency compared to participants in no-prime condition whose poststress latency was increased, *F*(1,68) = 3.03, *P* = .086.

#### 3.7.3. P300 Amplitude

No effects were detected.

#### 3.7.4. P300 Latency

A trend for time × prime interaction, *F*(1,66) = 4.13, *P* = .046, was detected. Though participants receiving prime showed a decrease in P300 latency and those receiving no prime showed an increase in P300 latency poststress compared to baseline, these differences were not significant.

### 3.8. Relationships among GNG, ERP, and Subjective Measures

Table 4 presents the relationships among GNG, ERPs, and subjective measures. As expected, ERP components were related: N200 amplitude was positively related to P300 amplitude, *r* = 0.55, *P* = .005, and no-go N200 and P300 latencies were also correlated, *r* = 0.995, *P* < .001. Poststress GNG measures and ERPs were not related.

Baseline expectation of aromatherapy effectiveness was related negatively to poststress no-go P300 amplitude, *r* = −0.44, *P* = .03, and positively to poststress alertness level, *r* = 0.44, *P* = .02. Alertness level at poststress assessment related negatively to median RT on GNG go trials, *r* = −0.35, *P* = .08, but this relationship did not reach conventional significance level.

Subjective stress ratings following stress battery did not correlate with any of the behavioral or ERP variables (data not shown).

## 4. Discussion

Our study is among the few investigations to date that evaluated effects of aromatherapy using a rigorous design with objective measures, adequate controls, and assessor blinding. In agreement with previous reports indicating expectancy as a part of the mechanism underlying aromatherapy benefits [18, 19], our results point to the importance of expectancy in aromatherapy actions. Specifically, our results showed that regardless of the experienced aroma a suggestion about powerful stress-reducing properties of aromatherapy benefits performance on a cognitive task subserved by higher-order cognitive functions. Furthermore, our data show differences between prime and no-prime conditions in ERP response inhibition indices indicative of protective effects of the prime on cognitive function.

### 4.1. Behavioral Results

Few errors of commission and omission for GNG in our study indicate that the task was easy for the participants. Easy behavioral tasks often fail to differentiate the performances in different groups or over time when healthy adults are tested [23]. However, when median RT was examined, participants receiving a prime prior to aromatherapy regardless of the type of aroma had a greater decrease in their median RT after exposure to stress than their nonprimed peers. These results suggest that increasing expectancy is beneficial for GNG performance and can potentially influence general attention and response inhibition processes. Our results agree with previous research showing that performance on tasks where reaction time is crucial can be affected by manipulating expectancy of improvement. For example, increasing expectancy of cognitive improvement by supplying placebo pills labeled as cognitive enhancer benefitted performance on several cognitive tasks including the choice reaction time task [36]. Due to simplicity of the go/no-go task in our study, there was no effect of increased speed on accuracy. However, assessing how accuracy of responding is affected by speed is warranted in future studies to clarify underlying mechanisms associated with expectancy for cognitive tasks performance.

### 4.2. ERP Results

We focused primarily on no-go N200 and P300 components representing subprocesses of response inhibition [41, 42] previously shown sensitive to stress [24]. These components are likely subserved by prefrontal areas [43, 44] and anterior cingulate cortex [45–47] considered vulnerable to acute stressors [48]. The study stress battery successfully elicited a stress response in participants as evidenced from a poststress increase in subjective and objective changes [21]. Subtle stress effects were also evident in ERP components for the whole sample. Specifically, poststress amplitudes tended to be lower than baseline amplitudes, likely reflecting negative stress effects. Further, because N200 of decreased amplitudes and longer latencies indicate poorer inhibitory control [26], our results suggest decreased poststress inhibitory control in our sample.

Our data show expectancy effect on N200 components as evidenced by divergent N200 amplitude for go and no-go trials between baseline and poststress depending on the prime subgroup. Suggesting powerful stress-reducing aromatherapy effects resulted in a largely preserved N200 profile in prime group compared to decreased poststress N200 amplitude in no-prime group. Because a decrease in N200 amplitude coupled with an increase in N200 latency points to poorer inhibition [26], receiving an expectancy-boosting prime might have prevented the subjects from experiencing decrements in poststress inhibitory control. This notion is further supported by an increase in poststress no-go N200 latency in those not receiving a prime in contrast to a decrease in this component in primed participants. Overall, patterns of no-go N200 indices in our sample indicate that enhancing aromatherapy expectancy regardless of actual aroma helps preserve prestress N200 profiles and benefits cognitive function after stress exposure.

The patterns observed for poststress N200 no-go latency paralleled those for poststress P300 no-go latency: increased P300 latency for no-prime group and decreased P300 latency for the prime group. Differences in P300 latency relate to stimulus classification speed with shorter latencies associated with superior cognitive performance [27]. Therefore, our results suggest that giving an expectancy-boosting prime before stress exposure benefits cognitive function. Some of the interesting ERP results in our study did not reach conventional significance level after adjustment for multiple comparisons; however, we believe the conclusions are valid because of a substantial agreement between behavioral and ERP data.

Interestingly, no significant aroma effects on ERP components were revealed potentially due to insufficient statistical power. There were also very few correlations among the study variables. Scarcity of correlations among subjective and objective stress-related variables has been previously reported and explained by complexity and variability of the components involved in producing a stress response. These components are believed to be dissociated due to different dynamics of the underlying systems [49]. Among the general lack of notable associations among cognitive performance, ERP, and subjective variables, it was encouraging seeing that preexisting expectation about aromatherapy positively related to poststress no-go P300 amplitude and poststress alertness level. This indicates that positive expectation of aromatherapy effects stemming from subject's personal experiences unrelated to experimental manipulation can also provide benefit.

Our results contribute to the growing evidence that verbally induced expectations can influence the therapeutic outcome, with the effects observable in active treatment and placebo groups [50]. More specifically, our findings are in agreement with several other studies reporting enhancement of cognitive performance and other cognition-related tasks, such as reaction times, by expectancies and placebos [36, 51–54]. Though traditionally expectancy and placebo effects have been treated as by-products of interventions and a nuisance in determining the treatment efficacy, more recently these effects have become the focus of investigations. Though not arising from an active procedure or agent, expectancy and placebo effects can produce a variety of effects through psychological and neurobiological mechanisms [55–57]. One of the most important mechanisms studied in relation to placebo responding is expectation or anticipation of therapeutic benefit, which is believed to influence health-related outcomes by reducing anxiety and activating the reward system [50]. Thus, though not arising from “real” treatment, the effects due to expectancy and placebo responses are real and beneficial to people who experience them, and therefore they are worth investigating. Understanding the role of expectancy and enhancing it when appropriate might optimize treatments and lead to better clinical outcomes.

Several limitations are important to mention with regard to our study. First, because our sample included generally healthy people between ages of 50 and 85, it is not clear how the present results generalize to other samples. Second, our sample was limited in size and aroma groups were smaller than subgroups based on the presence of the prime. Therefore, the sample might not have provided sufficient power to detect more subtle effects specific to aromatherapy. Studies assessing effects of expectancy and aromatherapy with larger samples and different populations are warranted. Next, expectancy of aromatherapy effect was assessed only at the baseline and no similar assessments were made after the prime or aroma exposure. Therefore, we cannot definitively state that the effects observed in the study were due to a changed expectancy in response to the prime statement. However, since all participants had the same procedure and the changes in wording of the instructions given prior to aroma exposure were the only difference between the prime and no-prime subgroups, we believe the differential outcome was due to that manipulation aimed at changing expectancy. Nevertheless, not directly assessing the change in expectancy after exposure to the prime is a limitation in our study. Further, the study was introduced as the assessment of different aromas for stress reduction without mentioning any placebo arms, and participants were not assessed at the end of the study on whether they thought they had an active stress-reducing aromatherapy or a placebo treatment. It might be beneficial to include an assessment of blinding success in any future studies. Despite these limitations, we believe our findings are valid and point to importance of expectancy in aromatherapy actions.

## 5. Conclusions

Overall, while not detecting aromatherapy-specific effect on stress, our study provides the evidence that manipulating expectancy by verbal suggestion of aromatherapy effectiveness might underlie some changes in behavior and brain function after exposure to aromatherapy. Our results indicate that enhancing beliefs in a positive effect of an intervention might be beneficial for performance.

## Figures and Tables

**Table 1:** Study timeline.

Baseline assessment	Stress battery	Poststress assessment
Self-reports	Cold pressor	Self-reports
Go/no-go task	IAPS	Go/no-go task
ERP	MIST	ERP

	Aromatherapy exposure	

**Table 2:** Comparisons of aroma groups and prime subgroups baseline characteristics.

Mean (SD) score unless otherwise noted	Aroma groups			*P* value	Prime groups		*P* value
	Lavender	Coconut	Water		Prime	No-prime	
	(*n* = 27)	(*n* = 27)	(*n* = 27)		(*n* = 40)	(*n* = 41)	
Age	59.1 (7.1)	57.5 (6.2)	56.5 (5.1)	.35	59.0 (6.2)	56.2 (5.9)	*.06 *
Female (%)	77.8	85.2	74.1	.59	82.5	75.6	.45
Education (years)	16.0 (2.1)	15.8 (1.9)	16.0 (3.2)	.93	15.3 (2.3)	16.6 (2.6)	**.04**
TICS score	38.5 (3.9)	39.2 (3.3)	37.0 (3.9)	.11	38.4 (3.6)	37.9 (4.0)	.60
PSS score	15.7 (5.1)	18.2 (5.0)	16.2 (6.1)	.28	17.0 (4.9)	16.4 (6.0)	.64
Previous aroma use (%)	55.6	40.7	42.3	.49	40.0	52.5	.26
Expected effectiveness	70.4 (13.5)	71.4 (13.0)	71.5 (12.1)	.95	71.4 (11.7)	70.8 (13.8)	.84
Expected stress change	33.6 (19.9)	33.2 (17.4)	34.4 (18.3)	.99	33.6 (17.5)	33.8 (19.4)	.99

Abbreviations: PSS = Perceived Stress Scale, SD = standard deviation, TICS = the Modified Telephone Interview for Cognitive Status.

Dependent Variables: Expected effectiveness = expected aromatherapy effectiveness for stress reduction, Expected stress change = expected change in stress level from neutral VAS score of 50 (less than 50 means decreased stress).

**(a):** 

Measures	Lavender		Coconut		Water	
	Baseline	Poststress	Baseline	Poststress	Baseline	Poststress
Go (% correct)	99.5 (0.9)	99.6 (0.8)	99.4 (0.8)	99.3 (1.3)	99.3 (1.4)	99.5 (1.2)
No-go (% correct)	84.5 (12.9)	87.5 (11.6)	78.4 (15.5)	81.5 (16.0)	83.7 (13.5)	85.9 (14.4)
Median RT, mean (SD)	366.3 (60.9)	346.9 (44.6)	354.3 (62.2)	334.7 (49.0)	355.2 (67.5)	342.1 (56.7)

SD = standard deviation.

Dependent variables: go = performance on go trials, no-go = performance on no-go trials, and median RT = median reaction time.

**(b):** 

Measures	Prime		No-prime	
	Baseline	Poststress	Baseline	Poststress
Go (% correct)	99.2 (1.3)	99.3 (1.3)	99.6 (0.6)	99.6 (0.9)
No-go (% correct)	81.9 (13.5)	84.3 (14.4)	82.6 (14.8)	85.8 (14.0)
Median RT, mean (SD)^a,b^	356.6 (59.6)	333.2 (48.2)	360.5 (66.9)	349.48 (51.0)

SD = standard deviation.

Dependent variables: go = performance on go trials, no-go = performance on no-go trials, and median RT = median reaction time.

^a^Prime × time interaction, *P* = .037; ^b^significant change in prime subgroup, *P* < .05.

**Table 4:** Relationships among expectancy, poststress behavioral, and poststress ERP measures.

Variable	2	3	4	5	6	7	8
(1) GNG med. RT	0.176	−0.054	0.051	−0.221	0.040	−0.082	−*0.346* ^T^
(2) GNG no-go % errors	1	−0.166	−0.309	−0.132	−0.278	0.304	0.151
(3) N200 no-go amplitude		1	0.138	**0.547** ^**^	0.124	−0.171	−0.165
(4) N200 no-go latency			1	−0.192	**0.995** ^**^	−0.288	−0.110
(5) P300 no-go amplitude				1	−0.189	**−0.441** ^*^	−0.147
(6) P300 no-go latency					1	−0.291	−0.106
(7) Expected aroma effect (baseline)						1	**0.443** ^*^
(8) Alertness level (poststress)							1

Abbreviations: GNG = go/no-go task, med. RT = median reaction time, SD = standard deviation, and ERP = event related potential.

^T^.10 < *P* < .05, ^*^ *P* < .05, and ^**^ *P* < .01.

## Abbreviations

EEG: Electroencephalography
ERP: Event related potentials
GNG: Go/no-go
IAPS: The International Affective Picture System
MIST: The Montreal Imaging Stressor Task
PANAS: The positive and negative affect schedule
PSS: Perceived stress scale
RT: Reaction time
SRT: Simple reaction time
TICS: The modified Telephone Interview for Cognitive Status
VAS: Visual analogue scale.
